# Decoupled Design for Highly Efficient Perchlorate Anion Intercalation and High‐Energy Rechargeable Aqueous Zn‐Graphite Batteries

**DOI:** 10.1002/advs.202306504

**Published:** 2023-12-08

**Authors:** Ying Zheng, Ting Deng, Xiaoyuan Shi, Hengbin Zhang, Bo Liu, Xun Li, Weitao Zheng

**Affiliations:** ^1^ Key Laboratory of Automobile Materials of MOE School of Materials Science and Engineering and Jilin Provincial International Cooperation Key Laboratory of High‐Efficiency Clean Energy Materials Jilin University Changchun 130012 China; ^2^ Key Laboratory of Polyoxometalate Science of Ministry of Education Northeast Normal University Changchun 130024 China; ^3^ Key Laboratory of Preparation and Application of Environmental Friendly Materials (Jilin Normal University) Ministry of Education Changchun 130103 China; ^4^ Chemical engineering department University of Chinese Academy of Science Beijing 100049 China

**Keywords:** anion intercalation, decoupled electrolytes, dual ion battery, graphite

## Abstract

Seeking new cathode chemistry with high onset potential and compatibility with electrolytes has become a challenge for aqueous Zn ion batteries. Anion intercalation in graphite (4.5 V vs Li^+^/Li) possesses the potentiality but usually shows a competitive relationship with oxygen evolution reaction (OER) in aqueous solutions. Herein, a decoupled design is proposed to optimize a full utilization of perchlorate ion intercalation in graphite cathode by pH adjustment. Benefiting from the decoupled design, high Coulombic efficiency is obtained by decelerating the kinetic of OER in acidic media. The decoupled Zn‐graphite battery exhibits a wide potential window of 2.01 V, as well as an attractive energy density of 231 Wh kg^−1^. In addition, a Zn‐graphite battery with ${\mathrm{SO}}_{4}^{2-}$ insertion is assembled, which demonstrates the capability of the proposed decoupled strategy to integrate novel electrode chemistries for high‐performance aqueous Zn‐based energy storage systems.

## Graphical abstract

An aqueous Zn‐graphite dual ion battery with efficient perchlorate anion intercalation in graphite cathode is presented. Based on a decoupled design, the parasitic oxygen evolution reaction is inhibited via pH regulation and electrolyte concentration, and wide potential windows and high energy density are achieved. The decoupled configuration also inspires more novel electrode chemistry combinations for advanced electrochemical energy storage.

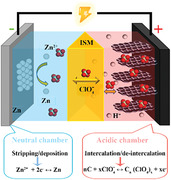

## 1 Introduction

**Figure 1:**
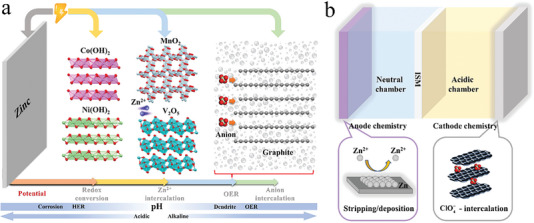
Working states of aqueous Zn ion batteries. a) Summary of typical cathode materials and challenges of aqueous Zn ion batteries. b) The decoupled design for an aqueous Zn ion battery, which is separated by an ion selected membrane into an acidic chamber for ${\mathrm{ClO}}_{4}^{-}$ (de)intercalation and a neutral chamber for Zn stripping/deposition.

By the virtues of high capacity and abundant reserves of zinc, incombustible aqueous Zn ion batteries (AZIBs) have become the spotlight in the pursuit of promising alternatives to Li‐ion batteries with lower cost and higher safety.^[^ ^1^, ^2^, ^3^ ^]^ Nevertheless, the inferior energy density limited by cathode materials with low redox potentials^[^ ^4^ ^]^ and aqueous electrolytes with narrow electrochemical stability window (ESW)^[^ ^5^ ^]^ has impeded their applications in large‐scale storage. For instance, the conversion potentials of Ni^2+^/Ni^3+^ and Co^2+^/Co^3+^ redox couples in alkaline media are 0.4 and 0.1 V (vs standard hydrogen electrode, SHE), which offer limited operating voltages for alkaline AZIBs (**Figure** **1**a).^[^ ^6^ ^]^ Although cathode materials, such as MnO_2_ and V_2_O_5_, can provide higher redox potentials in neutral media, the narrow ESW of dilute aqueous electrolytes still confine the cell voltage.^[^ ^7^, ^8^, ^9^, ^10^, ^11^ ^]^ Therefore, it is imperative to search for new cathode chemistries with high onset potentials and compatible electrolytes to achieve high energy density AZIBs.

Besides redox reaction and Zn^2+^ intercalation, anion intercalation in graphite occurs at higher potential (4.5 V vs Li^+^/Li, Figure 1b),^[^ ^12^, ^13^ ^]^ which as the cathode chemistry possesses the ability to achieve high operating voltage and energy density for AZIBs. However, the potential of anion intercalation in graphite usually exceeds the inherent thermodynamic oxidation of dilute aqueous solutions, namely oxygen evolution reaction (OER), which would result in low Coulombic efficiency (C.E.) and unsatisfactory lifespan. The concept of a “water‐in‐salt” electrolyte (WISE) can effectively enlarge ESW by reducing free water molecules in the electrolyte.^[^ ^14^, ^15^ ^]^ TFSI^−^, OTf^−^ or ${\mathrm{PF}}_{6}^{-}$‐based WISE have demonstrated their potentials in achieving wide operating windows for electrochemical energy storage devices in laboratory.^[^ ^16^, ^17^, ^18^ ^]^ But the cost raised by the high solute concentration in WISE is also should be taken into consideration towards practical applications. Thus, seeking intercalation ions with low cost and compatible electrolytes is essential for high‐performance AZIBs. Due to the chaotropic nature, high solubility and low cost, perchlorate ion (${\mathrm{ClO}}_{4}^{-}$) is deemed as a promising charge carrier for the intercalation chemistry. Compared to Li^+^, Na^+^ or Zn^2+^, perchlorate ion delivers a much lower hydration enthalpy (229 kJ mol^−1^), which affects the intercalation potential.^[^ ^19^ ^]^ Very recently, ${\mathrm{ClO}}_{4}^{-}$ (de)intercalation is utilized as the cathode chemistry in a prototype AZIB with a Zn(ClO_4_)_2_ WISE.^[^ ^20^ ^]^ The C.E. declines under 45% when a 2.6 V upper cut‐off voltage is set, which is probably due to the competitive relationship between ${\mathrm{ClO}}_{4}^{-}$ intercalation and OER and thus indicates that the cathodic potential limit of perchlorate salt WISE is still unsatisfied for ${\mathrm{ClO}}_{4}^{-}$ intercalation. On the other hand, the potential of OER depends on pH value of the solution. Theoretically, the kinetics of OER is more sluggish in acidic environment and thus requires higher onset potential, so that improving the sluggish kinetics of OER in acidic condition has become one of the top topics in water splitting.^[^ ^21^, ^22^ ^]^ Reversely thinking, decelerating the OER kinetics can be an effective method to facilitate ${\mathrm{ClO}}_{4}^{-}$ or other anion intercalation processes in aqueous electrolytes. But simultaneously, acidic environment is also corrosive for zinc anode and conducive to hydrogen evolution reaction (HER), which limits the output voltage, energy density and cyclability of AZIBs. Thus, designing better electrolyte and optimizing battery configuration held the key to address these issues.^[^ ^23^, ^24^ ^]^ Herein, a decoupled aqueous Zn ion battery (DAZIB) is proposed, in which ${\mathrm{ClO}}_{4}^{-}$ (de)intercalates in graphite in acidic condition while Zn^2+^ deposits/strips at Zn foil in neutral condition so as to enable an enlarged ESW (Figure 1b). The chemical crossover is prevented by an ion selected membrane (ISM) that separates the acidic chamber and neutral chamber. The OER kinetics is much retarded in acidic condition, which is demonstrated by a high C.E. and specific capacity of ${\mathrm{ClO}}_{4}^{-}$ (de)intercalation in graphite cathode. Experimental and computational analyses have been conducted to investigate the intercalation behavior, during which graphite intercalation compound (GIC) is reversibly formed. And in such a decoupled design, the assembled DAZIB exhibits a high upper cut‐off voltage of 2.65 V and a promising energy density of 231 Wh kg^−1^, with an outstanding cyclability with 81% capacity retention after 600 cycles. To demonstrate the high compatibility and feasibility of this decoupled design for AZIBs, another DAZIB with ${\mathrm{SO}}_{4}^{2-}$ intercalation in graphite cathode is also successfully assembled, which shows promising performance and also inspires us to discover more novel electrode chemistry combinations for advanced electrochemical energy storage.

## 2 Results and Discussion

**Figure 2:**
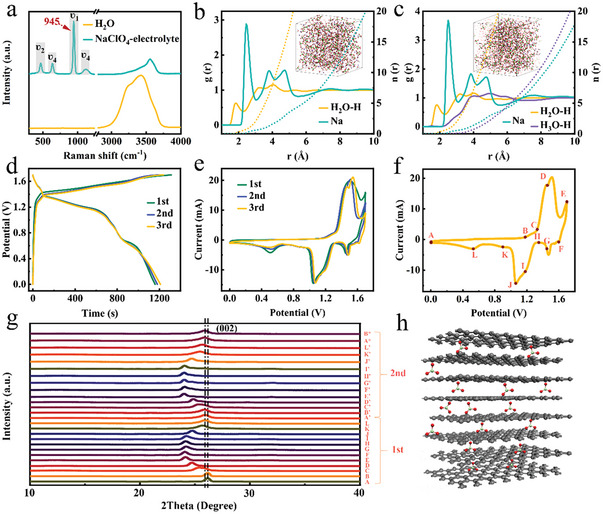
Electrochemical performance and corresponding structural evolution. a) Raman spectra of water and NaClO_4_ electrolyte. b) and c) The radial distribution function and coordinated number of NaClO_4_ and H^+^ + NaClO_4_ electrolyte. The dotted and the solid lines represent the coordinated number and the radial distribution function. d) Galvanostatic charge–discharge profiles of ${\mathrm{ClO}}_{4}^{-}$ intercalation in H^+^ + NaClO_4_ electrolyte of first three cycles at the current density of 250 mA g^−1^. e) CV curves of the first three cycles at the scan rate of 3 mV s^−1^. f) Sampling sites in the CV curve. g) In situ XRD analysis of graphite structure in first two cycles. h) ${\mathrm{ClO}}_{4}^{-}$ intercalation mode.

In intercalation chemistry, high content of charge carriers is desirable to maintain a high conductivity against the depletion of charge carriers during the intercalation into the host material. Therefore, a 9.435 m NaClO_4_ aqueous solution was utilized as the electrolyte (labeled as NaClO_4_ electrolyte), which was expected for a wide ESW. **Figure** **2**a displays the Raman spectra of water and NaClO_4_ solution. In the spectrum of water, a broad peak centered between 3000–3740 cm^−1^, belonging to H─O stretching vibration. In the NaClO_4_ electrolyte, this peak showed a blue shift, and the intensity was much reduced, indicating the activity of H_2_O was significantly reduced.^[^ ^17^ ^]^ Another four peaks were also observed in the NaClO_4_ electrolyte. The peaks of ν_1_ and ν_2_ correspond to the symmetrical stretching vibration and bending vibration of ${\mathrm{ClO}}_{4}^{-}$. And the peaks of ν_3_ and ν_4_ represent the asymmetrical stretching vibration and bending vibration of ${\mathrm{ClO}}_{4}^{-}$. In the unsolved ${\mathrm{ClO}}_{4}^{-}$, the ν_1_ peak is located at 930 cm^−1^, which was shifted to 945 cm^−1^ and indicated a solvation structure outside ${\mathrm{ClO}}_{4}^{-}$ in NaClO_4_ electrolyte.^[^ ^25^, ^26^ ^]^ The cyclic voltammetry (CV) curve of ${\mathrm{ClO}}_{4}^{-}$ (de)intercalation in graphite is depicted in Figure S1 (Supporting Information), which shows one pair of redox peaks between 1.3 and 1.7 V (vs saturated calomel electrode, SCE). The cathodic peak is very sharp and close to the upper cut‐off voltage so that the intercalation process is probably incomplete and accompanied with OER. And bubbles were observed at the surface of graphite in high potential range, evidencing the occurrence of OER (Figure S1, Supporting Information inset). Figure S2 (Supporting Information) displays the charge/discharge profiles of ${\mathrm{ClO}}_{4}^{-}$ (de)intercalation in graphite, whose discharge capacity is merely 36 mAh g^−1^ with a low C.E. of 21% indicating a fiercely competitive relationship between ${\mathrm{ClO}}_{4}^{-}$ intercalation and OER. The ESW of NaClO_4_ solution was evaluated by linear sweep voltammetry (LSV) measurement (Figure S3 Supporting Information), which shows a wide ESW of 2.34 V due to the depletion of water molecules in the solvation sheath in high concentration of salts. But the LSV line curling up at 1.36 V, at which potential ${\mathrm{ClO}}_{4}^{-}$ intercalation peak appeared, manifests that ${\mathrm{ClO}}_{4}^{-}$ intercalation potential is even beyond the ESW of NaClO_4_ electrolyte and OER is responsible for the low C.E. Accelerating the sluggish kinetics of OER in acidic condition is one of the top topics in the field of water splitting. Decelerating the OER kinetics, but on the contrary, is supposed to be an effective method to improve the domination of intercalation. Therefore, H^+^ was introduced into the electrolyte to inactivate OER. The color of the pH test paper was changed to dark purple, indicating the high acidity of H^+^ + NaClO_4_ electrolyte, and the cathodic limit was increased to 1.7 V correspondingly (Figure S4, Supporting Information). Theoretical simulations based on molecular dynamics were used to study the solvation structure of ${\mathrm{ClO}}_{4}^{-}$ in NaClO_4_ and H^+^ + NaClO_4_ electrolyte. The radial distribution function and coordinated number of H and Na in NaClO_4_ electrolyte are shown in Figure 2b. The calculated coordinated numbers of H and Na are 2.26 and 0.61, so the solvent structure of ${\mathrm{ClO}}_{4}^{-}$ in NaClO_4_ electrolyte can be expressed as Na_0.61_H_2_O_2.26_ClO_4_. In H^+^ + NaClO_4_ electrolyte, the coordinated numbers of H and Na are decreased to 2.13 and 0.56 (Figure 2c), suggesting the addition of H^+^ ions reduced the number of water molecules in the solvent sheath of ${\mathrm{ClO}}_{4}^{-}$. And the high density of H^+^ further inhibited OER from Equation (1):^[^ ^27^ ^]^

(1) $$2H_{2}O↔O_{2}+4H^{+}+4e^{-}$$

Figure 2d shows the charge/discharge profiles of the first three cycles in H^+^ + NaClO_4_ electrolyte. The flat charge/discharge plateaus are corresponding to ${\mathrm{ClO}}_{4}^{-}$ intercalation/deintercalation into/out of graphite. The discharge capacities are calculated to be 81, 82, and 85 mAh g^−1^ for the first three cycles, respectively. The C.E. of the first cycle is 88%, which is further increased to 95% for 2nd and 3rd cycle. Accordingly, the CV curves of ${\mathrm{ClO}}_{4}^{-}$ (de)intercalation become more complete in acidic condition compared to that in NaClO_4_ electrolyte (Figure 2e). One can see more than one pair of redox peaks in the CV curve of the 3rd cycle, implying both intercalation and deintercalation processes are stepwise. The cathodic peak of the 1st cycle is larger than the last two while the anodic peaks of these three cycles are almost identical, suggesting that more ${\mathrm{ClO}}_{4}^{-}$ intercalated into graphite cathode in the first cathodic scan. The Galvanostatic intermittent titration technique (GITT) test was applied to further evaluate the ${\mathrm{ClO}}_{4}^{-}$ diffusion kinetics (Figure S5, Supporting Information). The ${\mathrm{ClO}}_{4}^{-}$ diffusion coefficient ($D_{{\mathrm{ClO}}_{4}^{-}}$) in H^+^ + NaClO_4_ electrolyte is much higher than that in pure NaClO_4_ electrolyte, which suggests that acidic condition facilitates the intercalation by retarding OER process. The minimum $D_{{\mathrm{ClO}}_{4}^{-}}$ appears at the end of intercalation process, where ${\mathrm{ClO}}_{4}^{-}$ deeply intercalates into the graphite structure. And deintercalation gradually becomes slower as the $D_{{\mathrm{ClO}}_{4}^{-}}$ decreases in the process. To probe the ${\mathrm{ClO}}_{4}^{-}$ intercalation mechanism, in situ XRD experiments were conducted in first two cycles corresponding to the sampling site in Figure 2f. In the first cathodic scan, the (002) peak was widened and started to shift negatively at position C which is at the root of the cathodic peak, indicating the onset of intercalation (Figure 2g). And the potential of position C is also in accordance with appearance of plateaus in the charge process. The (002) peak gradually shifted from 26.14° to 24.13° from position A to E. Then, the (002) peak gradually recovered to 25.90° in the first anodic scan, which but was lower than the original position. Subsequently, the (002) peak shows a similar movement and the almost identical positions of A' and A^*^ prove a quite reversible (de)intercalation since the 2nd cycle. The gap between A and A' is probably because that a portion of ${\mathrm{ClO}}_{4}^{-}$ remained in graphite due to the decreasing $D_{{\mathrm{ClO}}_{4}^{-}}$ in the first deintercalation process, which expand the structure to facilitate following cycles (Figure 2h). In addition, (004) peak has a similar variation trend (Figure S6, Supporting Information), which also proves the ${\mathrm{ClO}}_{4}^{-}$ intercalation into graphite.

**Figure 3:**
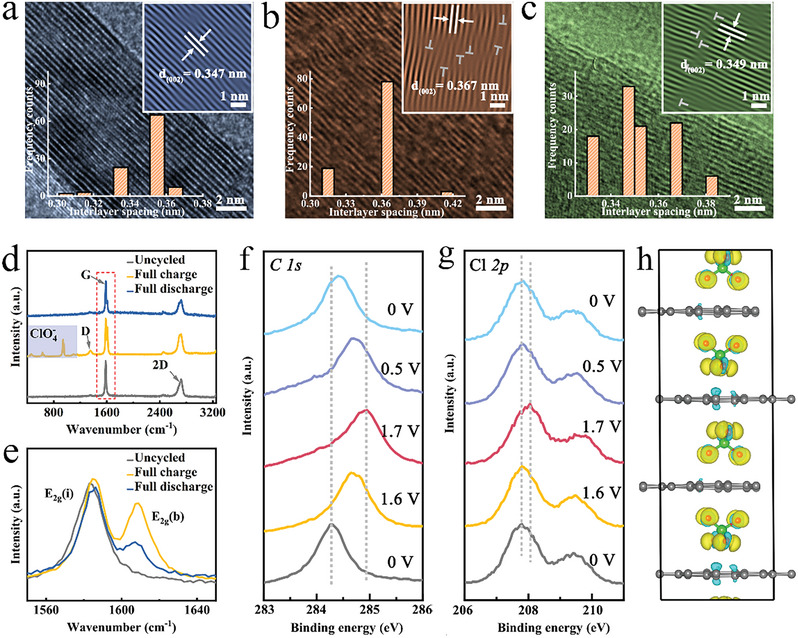
Structure conversion during charge/discharge cycles. a‐c) HRTEM images of pristine, full charge, and discharge graphite electrodes. Insets are calculated interlayer spacings. d) Raman spectra of the pristine, fully charged, and discharged graphite electrodes. e) Magnified Raman spectra of G band. f) and g) X‐ray photoelectron spectroscopy of C 1s and Cl 2p of graphite at various potentials. h) The charge density difference of ${\mathrm{ClO}}_{4}^{-}$ in graphite layers, and blue and yellow colors represent losing and accumulating electrons, respectively.

**Figure** **3**a displays the high‐resolution transmission electron microscope (HRTEM) image of the pristine graphite. The (002) plane of graphite can be clearly identified, and the lattice fringe was calculated as 3.47 Å. When the graphite was charged to 1.7 V in the first cycle, the layer distance was expanded to 3.67 Å (Figure 3b). And dislocations are also detected due to the stress induced by the intercalated ${\mathrm{ClO}}_{4}^{-}$ (Figure 3b inset). And the interlayer spacing was declined to 3.49 Å because of ${\mathrm{ClO}}_{4}^{-}$ deintercalation (Figure 3c). Therefore, based on the electrochemical results, in situ XRD analysis and TEM images, the first cycle can be regarded as an activation process, after which a portion of ${\mathrm{ClO}}_{4}^{-}$ remained in graphite structure to facilitate the subsequent cycles with higher efficiencies. To further analyze the behavior of ${\mathrm{ClO}}_{4}^{-}$ (de)intercalation in graphite, Raman experiments were conducted. Figure 3d shows the Raman spectra of the pristine, fully charged and discharged graphite electrodes. G and 2D bands^[^ ^28^ ^]^ located ≈1580 and 2700 cm^−1^ were detected in the pristine graphite electrode. No observation of D band indicates the high crystallinity of graphite electrode. When the graphite electrode was charged to 1.7 V, four peaks located within 400 to 1200 cm^−1^ belong to ${\mathrm{ClO}}_{4}^{-}$ appeared and D band was observed, implying that ${\mathrm{ClO}}_{4}^{-}$ has intercalated and induced defects in graphite structure.^[^ ^25^ ^]^ With closer scrutiny of G band (Figure 3e), it was divided into a high‐frequency component E_2g_(b) and a low‐frequency component E_2g_(i) in the fully charged graphite, which evidences the formation of GIC.^[^ ^29^, ^30^, ^31^, ^32^ ^]^ In addition, the intensity of 2D band was increased in the fully charged state, which relates to the number of graphite layers. Usually, graphene owns one high‐intensity 2D band than graphite because of fewer layers in graphene sheets.^[^ ^33^ ^]^ Thus, in our case, the enhanced 2D band suggests an enlarged interlayer spacing in graphite sheets due to ${\mathrm{ClO}}_{4}^{-}$ intercalation and GIC formation. When the graphite was discharged to 0 V, a weak E_2g_(b) component still existed. This phenomenon suggests the GIC residue after the first charge/discharge cycle, which is in accordance with forementioned electrochemical and in situ XRD analysis. Energy dispersive X‐ray spectroscopy mapping was also conducted to confirm the variation of elemental composition during ${\mathrm{ClO}}_{4}^{-}$ (de)intercalation. The samples we selected were the pristine graphite soaked in the electrolyte, fully charged and discharged graphite electrodes, in each of which we have sampled 100 sites to eliminate the occasionality. Figures S7 and S8 (Supporting Information) show the distribution of Cl and O in the graphite electrode. Low contents of Cl and O in the pristine graphite were observed due to the adsorption of electrolyte. When the graphite was charged to 1.7 V, the contents of Cl and O are higher than the pristine and discharged graphite. In addition, Cl and O contents in the fully discharged graphite are higher than the uncycled one, which is in consistency with aforementioned analysis and implies the GIC residue in the discharged graphite. To get more insight into ${\mathrm{ClO}}_{4}^{-}$ (de)intercalation, ex situ X‐ray photoelectron spectroscopy (XPS) experiments were conducted to identify the surface state changes during charge/discharge cycles. Figure 3f shows C 1s spectra of graphite electrode at different potentials. The prominent peak at 284.2 eV is the characteristic peak for carbon materials, which gradually shifted to higher position during perchlorate ion intercalation, and then shifted back in the deintercalation process. This reversible transformation suggests that the valence of C increased as ${\mathrm{ClO}}_{4}^{-}$ intercalating and declined during deintercalation. On the other hand, both Cl 2p and O 1s exhibited a similar transformation trend as C 1s (Figure 3g; Figure S9, Supporting Information), and the positive shifts of O 1s and Cl 2p suggest the oxidation of perchlorate ion.^[^ ^34^ ^]^ The charge density difference of ${\mathrm{ClO}}_{4}^{-}$ in graphite layers shows that both C and ${\mathrm{ClO}}_{4}^{-}$ partially lost parts of their electrons to form C_n_(ClO_4_)_x_‐GIC in the charge process (Figure 3h). The *π–π* network of graphite can be easily oxidized by ${\mathrm{ClO}}_{4}^{-}$ due to high electron affinity ability of Cl. The covalent nature of C─Cl bond indicates the formation of shared electrons in GIC compound, in which the shared electrons deviated from the equilibrium position. As a result, the valences of Cl and O are both increased.

**Figure 4:**
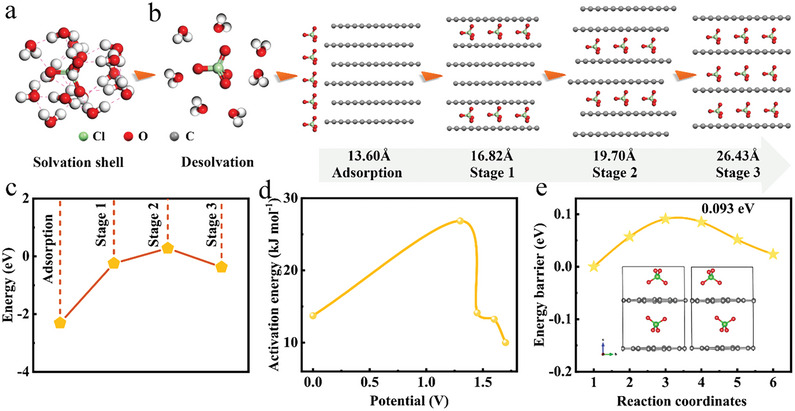
Theoretical investigations of ${\mathrm{ClO}}_{4}^{-}$ (de)intercalation mechanism. a) Solvation mode of ${\mathrm{ClO}}_{4}^{-}$ in the electrolyte. b) The ${\mathrm{ClO}}_{4}^{-}$ intercalation process that undergoes desolvation, adsorption and intercalation. c) The adsorption energy and intercalation energy of ${\mathrm{ClO}}_{4}^{-}$ intercalation for stages 1 to 3. d) The activation energy of ${\mathrm{ClO}}_{4}^{-}$ intercalation processes based on electrochemical impedance spectroscopy tests. e) The diffusion energy barrier of ${\mathrm{ClO}}_{4}^{-}$ intercalation in graphite layers.

In order to provide more insight into ${\mathrm{ClO}}_{4}^{-}$ intercalation behavior, we carried out a series of Gauss and VASP calculations based on the first principles. In aqueous electrolyte, water molecules surround electrolyte ions to form a hydration shell. We constructed a solvation mode of ${\mathrm{ClO}}_{4}^{-}$ in NaClO_4_ electrolyte, in which 15 H_2_O molecules can completely wrap one ${\mathrm{ClO}}_{4}^{-}$ ion (**Figure** **4**a). Inversely, there is a desolvation process to obtain isolated ${\mathrm{ClO}}_{4}^{-}$ before the adsorption and intercalation processes (Figure 4b). Table S1 (Supporting Information) summarizes the energy parameters of isolated ${\mathrm{ClO}}_{4}^{-}$ ion, hydrated ${\mathrm{ClO}}_{4}^{-}$ ion and water molecules. The solvation energy of ${\mathrm{ClO}}_{4}^{-}$ ion was calculated to be −2.31 eV. It is higher than the hydration energy of TFSI^−^ (−2.841 eV),^[^ ^17^ ^]^ which indicates that ${\mathrm{ClO}}_{4}^{-}$ has lower intercalating potential than TFSI^−^ ion. Figure S10 (Supporting Information) displays the adsorption of of ${\mathrm{ClO}}_{4}^{-}$ on edge plane of graphite, in which ${\mathrm{ClO}}_{4}^{-}$ ion was adsorbed on the edge through Cl atom in the central. And the adsorption energy value of ${\mathrm{ClO}}_{4}^{-}$ was calculated to be −2.92 eV. The top and main views of subsequent intercalation process with different stages are depicted in Figure S11 (Supporting Information). The plane of O atoms in ${\mathrm{ClO}}_{4}^{-}$ is almost parallel to the hexagonal carbon ring in stages 1 and 2. Due to the increasing ionic repulsion between ${\mathrm{ClO}}_{4}^{-}$ ions in adjacent layers as more into graphite interlayers, ${\mathrm{ClO}}_{4}^{-}$ rotated from stage 2 to 3 and diffused in such an angle. Accordingly, the structural change of graphite was also simulated during ${\mathrm{ClO}}_{4}^{-}$ intercalation, and the *a*, *b* and *c* parameters of graphite in different stages are summarized in Table S2 (Supporting Information). From adsorption to stage 3, the interlayer spacing (*c* direction) was gradually enlarged as ${\mathrm{ClO}}_{4}^{-}$ intercalated into graphite, while the structure along *a* and *b* directions sightly decreased to counteract the stress induced by the growth in *c* direction. The calculated intercalation energies of ${\mathrm{ClO}}_{4}^{-}$ in different stages are shown in Figure 4c: −2.92, −0.24, 0.28 and −0.37 eV for adsorption, stage 1, stage 2 and stage 3, respectively. Stage 2 shows the highest intercalation energy than other stages. Correspondingly, we also calculated the activation energy at different potentials in one charge based on a series of electrochemical impedance spectroscopy tests (Figure 4d; Figure S12, Supporting Information). The activation energy represents the energy barrier where perchlorate was intercalated into graphite to form C_n_(ClO_4_)_x_‐GIC. The activation energies of charging process are: 13.72, 26.85, 14.14, 13.23, and 10.01 kJ mol^−1^ for different potentials, respectively, which exhibits the same variation trend as the simulated ${\mathrm{ClO}}_{4}^{-}$ intercalation energy. The static repulsion between ${\mathrm{ClO}}_{4}^{-}$ ions became stronger as more ${\mathrm{ClO}}_{4}^{-}$ intercalating into graphite, which increases the intercalation energy of subsequent ${\mathrm{ClO}}_{4}^{-}$ ions. And the decline of the activation and intercalation energies is probably because of the increased graphite layer spacing. In addition, the density of state of GIC at different stages show fewer electronic states across the Fermi level, suggest a decreasing conductivity as ${\mathrm{ClO}}_{4}^{-}$ intercalate into graphite which thus requires more intercalation or activation energy to continue the intercalation (Figure S13, Supporting Information).^[^ ^35^ ^]^ To our surprise, the energy barrier of ${\mathrm{ClO}}_{4}^{-}$ diffusing to next position in graphite matrix is only 0.093 eV (Figure 4e and Figure S14, Supporting Information), which may facilitate a fast diffusion process during the electrochemical process.

Based on aforementioned analysis, the reaction of ${\mathrm{ClO}}_{4}^{-}$ (de)intercalation can be interpreted as Equation (2):

(2) $$\mathrm{nC}+{\mathrm{xClO}}_{4}^{-}↔{\;C}_{n}{\left({\mathrm{ClO}}_{4}\right)}_{x}+{\mathrm{xe}}^{-}$$

**Figure 5:**
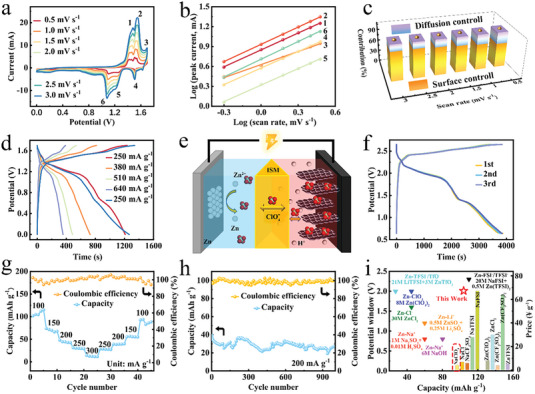
Electrochemical evaluations of ${\mathrm{ClO}}_{4}^{-}$ (de)intercalation in the Zn‐graphite battery. a) CV curves of ${\mathrm{ClO}}_{4}^{-}$ (de)intercalation at different scan rates. b) log (*i*) versus log (*v*) plot at peak current and *b* values of four peaks by linear fit. c) Surface pseudocapacitive contribution at different scan rates. d) Charge/discharge profiles at different current densities. e) The decoupled configuration of the Zn‐graphite battery. f) The charge/discharge profiles of the first three cycles of decoupled aqueous Zn ion battery at the current of 100 mA g^−1^. g) Rate capability and corresponding Coulombic efficiency of decoupled aqueous Zn‐ion battery. h) Cyclability at the current density of 200 mA g^−1^. i) The comparison of the potential window between this work and recently reported aqueous Zn‐ion batteries.

The electrochemical properties of ${\mathrm{ClO}}_{4}^{-}$ (de)intercalation in the graphite cathode were systematically analyzed in a three‐electrode system with H^+^ + NaClO_4_ electrolyte, in which graphite, platinum plate and calomel saturated electrode were utilized as working, counter and reference electrodes, respectively. Figure S15 (Supporting Information) exhibits the CV curves at the scan rate of 2 mV s^−1^ with different cut‐off voltage, in which we can see that the intercalation process initiated at 1.4 V. The higher the voltage reaches, the more prominent and complete redox peaks are, indicating a high level of ${\mathrm{ClO}}_{4}^{-}$ intercalation in graphite. **Figure** **5**a shows the CV curves of graphite at different scan rates with the cut‐off potential of 1.7 V and three pairs of redox peaks manifest that both ${\mathrm{ClO}}_{4}^{-}$ (de)intercalation processes are stepwise. As the scan rate increased, no shift of cathodic and anodic peaks was observed, suggesting the good reversibility of ${\mathrm{ClO}}_{4}^{-}$ (de)intercalation. The (de)intercalation reaction kinetics were analyzed to investigate whether the charge/discharge processes are surface or diffusion‐controlled. Figure 5b presents the plots of log(*i*) versus log(*v*) from 0.5 to 3 mV s^−1^ for these three pairs of redox peaks, in which the scan rate and corresponding current were studied by the following Equation (3):^[^ ^36^ ^]^

(3) $$i={\mathrm{av}}^{b}$$

where *a* and *b* are two fitted values that provide the information on the dominant mechanism. When the *b* value is 1, the current is surface‐controlled. If the *b* value reaches 0.5, then the current is diffusion‐controlled. The fitted *b* values of the cathodic peaks of ${\mathrm{ClO}}_{4}^{-}$ intercalation are 0.85, 0.87, and 0.66, implying a gradual transformation from surface to diffusion‐controlled mechanism in ${\mathrm{ClO}}_{4}^{-}$ intercalation. While *b* values of the anodic peaks are 0.83, 0.83, and 0.89, indicating that both processes contribute to discharge capacity. The diffusion‐controlled and surface‐controlled capacities can be deconvoluted based on the CV curves and following Equation (4):^[^ ^37^ ^]^

(4) $$i=k_{1}v+k_{2}v^{\left(1/2\right)}$$

in which k_1_v and k_2_v^(1/2)^ stand for the surface‐ and diffusion‐controlled process, respectively. As the scan rate increased from 0.5 to 3 mV s^−1^, the surface‐controlled capacities are 68%, 73%, 76%, 79%, 81%, and 81%, respectively. (Figure 5c). We also analyzed the capacity contribution of the surface‐controlled process at different upper cut‐off voltages to see the intercalation degree. Figures S16–S20 (Supporting Information) exhibit the fractions of surface‐controlled contribution with different upper cut‐off voltages. The fractions of surface contributions are 63%, 66%, 64%, 64%, 66%, and 68% as the cut‐off voltage rising from 1.2 to 1.7 V, which are quite stable and imply a high respond speed. Figure 5d displays the charge/discharge profiles at the cut‐off voltage of 1.7 V. Flat charge/discharge plateaus was observed at different current densities, which evidences the ${\mathrm{ClO}}_{4}^{-}$ (de)intercalation behavior. The specific capacity was calculated to be 86 mAh g^−1^ at the current density of 250 mA g^−1^. And the specific capacity reaches 78, 67, and 63 mAh g^−1^ when the current density was increased to 380, 510, and 640 mA g^−1^, respectively. A specific capacity of 89 mAh g^−1^ is obtained when the current density returns to 250 mA g^−1^. The higher capacity was because the first cycle can be deemed as an activation process in which ${\mathrm{ClO}}_{4}^{-}$ ions residue remained in the graphite to facilitate subsequent cycles (Figure S21, Supporting Information). After 100 cycles at the current density of 510 mA g^−1^, a specific capacity of 53 mAh g^−1^ was obtained which proves a good cyclability (Figure S22, Supporting Information).

The deceleration of OER kinetics by reducing pH favors the ${\mathrm{ClO}}_{4}^{-}$ intercalation at high potential. However, the overall right shift of LSV of the acidic electrolyte reduces the operation window of an anode. In addition, acidic media would also trigger serious corrosion reactions and HER at the Zn anode. Correspondingly, a DAZIB was proposed, which is schematically illustrated in Figure 5e. The DAZIB consists of acidic and neutral chambers for graphite cathode and Zn anode which was separated by an ISM. The anode side contains a zinc foil in a Zn^2+^ + NaClO_4_ anolyte, while a graphite electrode in a H^+^ + NaClO_4_ catholyte is utilized as the cathode to suppress electrolyte decomposition. Cathode and anode reactions take place in the decoupled environment, as shown in Equations (5) and (6):

(5) $$\mathrm{Cathode}:\;\mathrm{nC}+{\mathrm{xClO}}_{4}^{-}↔C_{n}{\left({\mathrm{ClO}}_{4}\right)}_{x}+{\mathrm{xe}}^{-}$$

(6) $$\mathrm{Anode}:{\mathrm{Zn}}^{2+}+2e^{-}↔\mathrm{Zn}$$

In the charge process, ${\mathrm{ClO}}_{4}^{-}$ intercalates into graphite structure and Zn^2+^ deposits on Zn anode. Inversely, ${\mathrm{ClO}}_{4}^{-}$ de‐intercalates out of graphite layers and Zn is simultaneously oxidized to Zn^2+^ to release energy in the discharge process. Figure S23 (Supporting Information) exhibits the CV curves of DAZIB at the scan rate of 3 mV s^−1^ with a high up cut‐off voltage of 2.65 V. And charge/discharge profiles of first three cycles of DAZIB are displayed in Figure 5f. The discharge capacity reaches 102, 105, and 104 mAh g^−1^ at the current density of 100 mA g^−1^ with a high C.E. of 102%, 100%, and 98%, respectively. As the discharge current density increase from 100 to 300 mA g^−1^, the capacities are calculated to be 104, 74, 48, 32, and 15 mAh g^−1^ with high C.E. (97–100%, Figure 5g), respectively. The reason for this excellent rate capability is twofold: 1) The low diffusion barrier of ${\mathrm{ClO}}_{4}^{-}$ in graphite endows it with fast kinetics. 2) The decoupled configuration possesses the shuttle advantage of dual ion battery, in which the diffusion distance of charge carrier is halved so as to enable an excellent rate capability. The maximum energy density is calculated to be 231 Wh kg^−1^. After 600 cycles at the current density of 200 mA g^−1^, the capacity retention reaches 81%, indicating a stable cyclability (Figure 5h). Compared to recently reported aqueous Zn dual ion batters with anion intercalation cathodes,^[^ ^20^, ^38^, ^39^, ^40^, ^41^, ^42^, ^43^ ^]^ the DAZIB with ${\mathrm{ClO}}_{4}^{-}$ intercalation can provide promising electrochemical performance but with much‐reduced cost by using cheap NaClO_4_ instead of expensive salts such as LiTFSI, NaFSI or Zn(ClO_4_)_2_ or reducing electrolyte concentration (Figure 5i). On the other hand, this DZAIB also shows a higher potential window and thus higher energy density than recent aqueous Zn dual ion batteries with cation intercalation cathodes, which acknowledges the advantages of the proposed decoupled strategy.

Usually, onset potential of intercalation chemistry is higher than that of conversion and alloying reactions, which thus can provide higher output potential windows and eventually higher energy densities. In aqueous system, the high intercalation potential may be inapplicable due to the narrow voltage window of water (1.23 V), suppressing the operating voltage and leading to unsatisfying energy density. A few reports have shown the high intercalation potential of ${\mathrm{ClO}}_{4}^{-}$ in graphite in aqueous systems. Nevertheless, as the upper cut‐off voltage increased, the specific capacity exhibited an exponential growth whereas C.E. dropped rapidly, suggesting the concurrence of ${\mathrm{ClO}}_{4}^{-}$ intercalation and OER. Combining the experimental and computational results, we have investigated the (de)intercalation behavior of ${\mathrm{ClO}}_{4}^{-}$ in graphite as the cathode chemistry for aqueous Zn battery, which turns out to be quite promising after breaking through the OER barrier in a decoupled system. This decoupled system has successfully decelerated OER kinetics, which alleviates the competitiveness of ${\mathrm{ClO}}_{4}^{-}$ intercalation. In addition, the decoupled configuration is also immune to dramatic hydrogen evolution reactions and serious corrosion of the Zn anode in acidic media. As a result, the specific capacity and energy density of this DAZIB have been doubled than other reports of ${\mathrm{ClO}}_{4}^{-}$ intercalation. On the other hand, such a decoupled configuration also provides an opportunity to search for more cathodes with the intercalation chemistry for high working potential, C.E. and capacity. To demonstrate the feasibility of this concept, another DAZIB with ${\mathrm{SO}}_{4}^{2-}$ insertion and dilute electrolyte was assembled (Figure S24, Supporting Information), which delivered a potential window of 1.925 V and an energy density of 40 Wh kg^−1^ at 100 mA g^−1^ (Figure S25, Supporting Information). These DAZIBs have proved that this decoupled configuration not only widens the operating window of aqueous Zn battery but also provides a high compatibility of novel electrode chemistry combinations for high‐performance aqueous energy storage systems.

## 3 Conclusion

The ${\mathrm{ClO}}_{4}^{-}$ intercalation mechanism in graphite was systematically studied by theoretical simulation, in situ and ex situ characterizations, which shows high intercalation potential, fast diffusion speed and high specific capacity in acidic media by decelerating the OER kinetics in acidic media. Herein, A novel decoupled Zn‐graphite is successfully assembled with high C.E., specific capacity and cyclability. The separated configuration enables a high utilization of ${\mathrm{ClO}}_{4}^{-}$ intercalation in graphite cathode in acidic media, providing a high energy density of 231 Wh kg^−1^. After 600 cycles, a high energy retention of 81% is observed, which benefits from this decoupled design that prevents the competition between OER and ${\mathrm{ClO}}_{4}^{-}$ intercalation. The excellent properties of the assembled DAZIBs demonstrate the high feasibility of this decoupled strategy for more novel electrode chemistry combinations and the potential to realize high‐performance aqueous energy storage systems for the next generation.

## 4 Experimental Section

### Materials and Chemical

Commercial graphite paper and the high‐purity Zn foil as the electrode were purchased from Sinosteel Shanghai New Graphite Material Co., LTD and Canrd Corporation, respectively. Zn(CF_3_SO_3_)_2_, NaClO_4,_ and ZnSO_4_ were purchased from Aladdin Reagent Co., LTD and Sinopharm Chemical Reagent Co., LTD. The HClO_4_ and H_2_SO_4_ with mass fractions of 70% and 95% were purchased from Tianjin Xinyuan Chemical Co., LTD. AMV and AMI 7001s anion selected membrane as the separator were purchased from AGC Engineering Co., LTD and Functional Membranes and Plant Technology, respectively.

### Physical Characterization

The in situ XRD tests were performed on a Bruker D8 Advance diffractometer with Cu Kα radiation (*λ* = 0.15 418 nm). Thermo ESCALAB 250Xi and Thermo DXR2 (325 nm) devices were used for XPS and Raman test. The HRTEM image is obtained by FEI Talos F200 device. The energy dispersive X‐ray spectroscopy equipped with Hitachi SU‐8010 scanning electron microscope was used for element content analysis, and each of the pristine, charged, and fully discharged carbon papers was sampled 100 times to eliminate occasionality.

### Electrochemical Characterization

The galvanostatic charge–discharge, LSV, andCV tests were carried out by an electrochemical workstation (Shanghai Chenhua 660E). Two platinum plate electrodes were used as the working electrode and the counter electrode during the LSV test. The Ivium and Donghua DH7000C electrochemical workstations were used to performGITT and electrochemical impedance spectroscopy tests, respectively. The cathode was charged/discharged for 30 s at the current of 2 mA and maintained for 600 s when the current was 0 mA in the GITT test. The diffusion coefficient D can be calculated according to the following Equation (7):

(7) $$D=\frac{4}{\pi \tau }{\left(\frac{n_{m}V_{m}}{S}\right)}^{2}{\left(\frac{\Delta E_{s}}{\Delta E_{t}}\right)}^{2}$$

The τ is the relaxation time. The *n*_m_, *V*_m_, and *S* are the molar number, molar volume, and electrode/electrolyte contact area, respectively. The electrochemical test of a single electrode was carried out in a three‐electrode system. Graphite paper, calomel saturated electrode and a platinum plate were used as the working, reference, and counter electrode, respectively. The 9.435 m NaClO_4_ electrolyte was obtained by dissolving 31.8087 g NaClO_4_ in 9.24492 g deionized water and labeled NaClO_4_ electrolyte. The H^+^ + NaClO_4_ electrolyte was acquired by adding 8 ml HClO_4_ (25 vol%) to the previous NaClO_4_ electrolyte. The specific capacity is calculated according to the following Equation (8):

(8) $$C_{m}=\frac{I_{m}\times t}{3600}$$

in which *C*_m_ represents the mass‐specific capacity (mAh g^−1^), *I*_m_ current density, (mA g^−1^), *t* the discharging time (s), respectively. The Zn‐graphite battery was tested in a two‐electrode system. Graphite paper, zinc foil, H^+^ + NaClO_4_, and Zn^2+^ + NaClO_4_ (2 m Zn(CF_3_SO_3_)_2_ + 9.435 m NaClO_4_) solutions were utilized as cathode, anode, catholyte, and anolyte, respectively. The two chambers were separated with an AMV anion‐selected membrane. Similarly, another decoupled aqueous Zn‐ion battery with ${\mathrm{SO}}_{4}^{2-}$ insertion was assembled by using graphite paper and zinc foil as the cathode and anode and 7 m H_2_SO_4_ and 2 m ZnSO_4_ solutions as the catholyte and anolyte, respectively. The two chambers were separated by an AMI 7001s anion‐selected membrane. The energy and power density of the full battery was calculated according to the following Equations (9) and (10):

(9) $$E_{m}=\frac{I\times V\times t}{m}$$

(10) $$P_{m}=\frac{E_{m}}{t}$$

Where *E*_m_ and *P*_m_ are energy density (Wh kg^−1^) and power density (W kg^−1^), respectively. *I*, *V*, and *t* represent current (A), voltage (V) and discharge time (s), respectively.

### Computational Method

The graphite supercell model was constructed and calculated using the Vienna Ab Initio Package (VASP) and density functional theory.^[^ ^44^, ^45^ ^]^ The Perdew‐Burke‐Ernzerhof formula was used in the generalized gradient approximation.^[^ ^46^ ^]^ The van der Waals interaction was calculated using the empirically corrected DFT + D3 method in Grimme's scheme. The projected augmented wave potential was chosen to describe ionic nuclei and to take valence electrons into account using a plane‐wave basis set with a kinetic energy cutoff of 450 eV.^[^ ^47^ ^]^ When the energy change was <10^−5^ eV, the electron energy self‐consistency was satisfied, and when the energy change was <0.02 eV Å^−1^, the geometric optimization was considered to be convergent. The vacuum spacing perpendicular to the plane of the structure is 18 Å when surface calculations are performed. Finally, the adsorption energy and intercalating energy are obtained according to following Equations (11) and (12):

(11) $$E_{\mathrm{ad}}=E\left(C_{n}-{\mathrm{ClO}}_{4}^{-}\right)-E\left(C_{n}\right)-E\left({\mathrm{ClO}}_{4}^{-}\right)$$

(12) $$E_{\mathrm{int}}=\frac{E\left(C_{n}{\left[{\mathrm{ClO}}_{4}^{-}\right]}_{m}\right)-E\left(C_{n}\right)-m\;E\left({\mathrm{ClO}}_{4}^{-}\right)}{m}$$

Where *E*_ad_ and *E*_int_ are adsorption and intercalating energy, respectively. E(C_n_–${\mathrm{ClO}}_{4}^{-}$), E(C_n_), E(${\mathrm{ClO}}_{4}^{-}$) and E(C_n_[${\mathrm{ClO}}_{4}^{-}$]_m_) are the energies of graphite with ${\mathrm{ClO}}_{4}^{-}$ adsorbed on the edge, pure graphite structure, ${\mathrm{ClO}}_{4}^{-}$ and ${\mathrm{ClO}}_{4}^{-}$ intercalated graphite, respectively. The *m* is the number of intercalated ${\mathrm{ClO}}_{4}^{-}$. When calculating the migration energy barrier, the ${\mathrm{ClO}}_{4}^{-}$ is moved to another structure with equivalent adsorption position, and then the CI‐NEB method is used to find the transition state, and the CI‐NEB energy curve of the migration process is obtained. Gaussian 16 combined with B3LYP‐D3BJ/6‐31 + G(d,p)/SMD(water) was used to optimize the hydration structure,^[^ ^48^ ^]^ and B3LYP‐D3BJ/6‐311 + G(d,p)/SMD(water) was used to calculate the hydration energy.^[^ ^49^ ^]^

The structures of NaClO_4_ and H^+^ + NaClO_4_ electrolytes were calculated by molecular dynamics the Molecular Dynamics simulations using the Gromacs program suite^[^ ^50^ ^]^ with the hybridized force field of OPLS‐2009IL and Madrid‐2019 force fields.^[^ ^51^, ^52^ ^]^ The ${\mathrm{ClO}}_{4}^{-}$ and Na^+^ ions cations were parameterized using OPLS‐2009IL and Madrid‐2019 forcefields, respectively. The H_2_O was simulated using the OPC3 parameter. The forcefield parameters of H_3_O^+^ were generated using the Seminario method^[^ ^53^ ^]^ and the RESP charge model. All these topology files of these molecules and ions were generated directly using AuToFF web server. The initial simulation boxes were established using the PACKMOL program with dimensions of 6 × 6 × 6 nm^3^.^[^ ^54^ ^]^ First, the structures were energy‐minimized and then annealed from 0 to 298.15 K over a 1 ns time period and time step is 1 ps to reach an equilibrium state. The temperature was maintained at 298.15 K by the velocity‐rescale thermostat with a relaxation constant of 1 ps.^[^ ^55^ ^]^ The pressure was maintained at 1.01325 × 10^5^ Pa using Berendsen's barostat with an isothermal compressibility constant of 4.5 × 10^−5^. Periodic boundary conditions were applied in all directions and the electrostatic interactions and van der Waals forces were treated using the Particle‐mesh Ewald (PME) method with a cut‐off distance of 15 Å. A 20 ns MD simulation was carried out following the energy‐minimization and equilibration steps, while the trajectory saved every 1 ps. The results were analyzed using Gromacs tool suites and the Visual Molecular Dynamic program.^[^ ^56^ ^]^

### Statistical Analysis

The illustration models were established using VESTA software (National Museum of Nature and Science, Japan) and Office software (Microsoft, America). XRD, Raman, HRTEM and XPS data were analyzed using Jade software (Materials Data, America OriginPro software (Origin Lab, America), Digital Micrography software (Gatan, Inc., America) and Avantage software (Thermo Fisher Scientific, America), respectively. Cyclic voltammetry, Linear sweep voltammetry, Galvanostatic charge–discharge data were analyzed by CHI 660E software (CH Instruments, Inc., America). The Galvanostatic intermittent titration technique and electrochemical impedance spectroscopy were analyzed by Ivium software (Ivium Technologies B.V., Netherlands) and ZView software (Scribner, LLC, America), respectively. The theoretical calculation results of the electrolytes were analyzed by Visual Molecular Dynamics software, while the data of the electrodes were analyzed by VESTA software. Finally, analyzed data was drawn using OriginPro software and merged with Adobe Photoshop software (Adobe Systems Inc., America).

## Conflict of Interest

The authors declare no conflict of interest.

## Supporting information

Supporting Information

## Data Availability

The data that support the findings of this study are available from the corresponding author upon reasonable request.
