# Supplementary material for: Decoupled Design for Highly Efficient Perchlorate Anion Intercalation and High‐Energy Rechargeable Aqueous Zn‐Graphite Batteries
Source: Adv Sci (Weinh). 2023 Dec 8;11(7):2306504. doi: 10.1002/advs.202306504 (PMC10953716; doi:10.1002/advs.202306504)
Supplement: Supplementary file 1 — Supporting Information [file ADVS-11-2306504-s001.pdf]

ADVANCED SCIENCE
Open Access

Supporting Information

for *Adv. Sci.*, DOI 10.1002/advs.202306504

Decoupled Design for Highly Efficient Perchlorate Anion Intercalation and High-Energy Rechargeable Aqueous Zn-Graphite Batteries

Ying Zheng, Ting Deng, Xiaoyuan Shi, Hengbin Zhang, Bo Liu, Xun Li and Weitao Zheng**

Supporting Information

Decoupled Design for Highly Efficient Perchlorate Anion Intercalation and High-energy Rechargeable Aqueous Zn-graphite Batteries

Ying Zheng, Ting Deng, * *Xiaoyuan Shi, Hengbin Zhang, Bo Liu, Xun Li, and Weitao Zheng**

Figure S1-S25 & Table S1-S2

Figures:

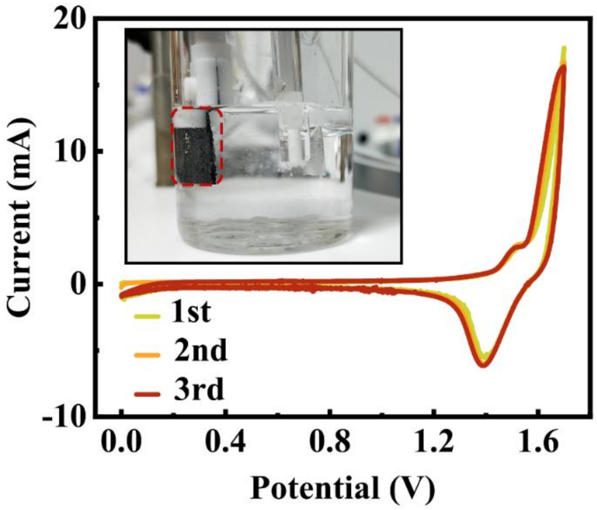

Figure S1. Cyclic voltammetry curves of ClO_4^- (de)intercalation in graphite in $NaClO_4$ electrolyte in first three cycles. Scan rate: 3 mV s^{-1}.

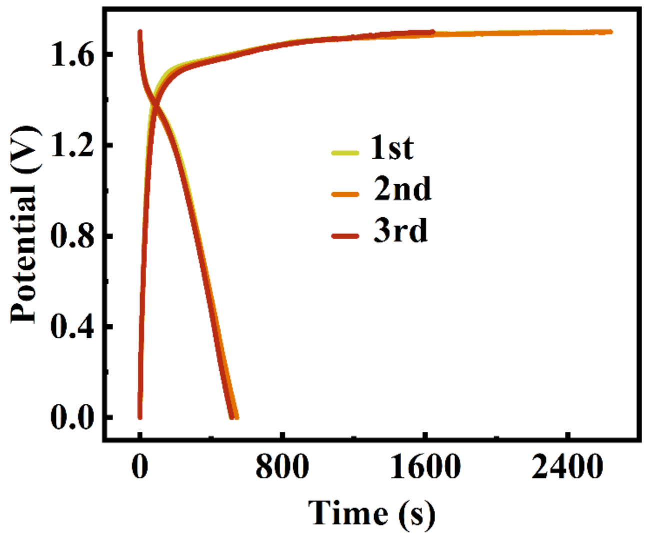

Figure S2. The galvanostatic charge-discharge profiles of ClO_4^- (de)intercalation in graphite in $NaClO_4$ electrolyte in first three cycles. Current density: 250 mA g^{-1}.

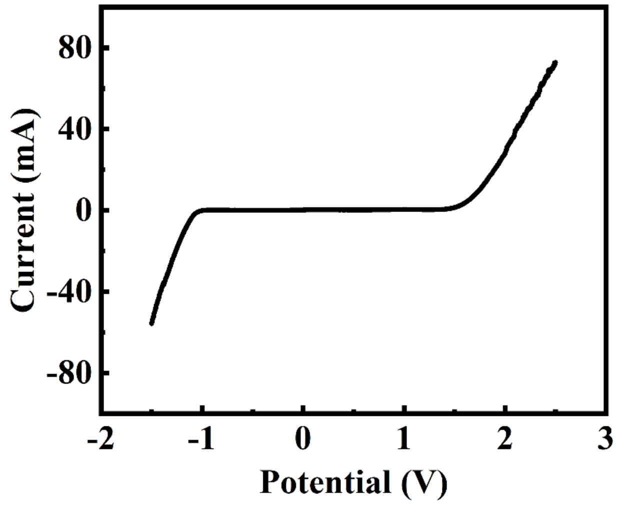

Figure S3. Linear sweep voltammetry of $NaClO_4$ electrolyte. Scan rate: 5 mV s^{-1}.

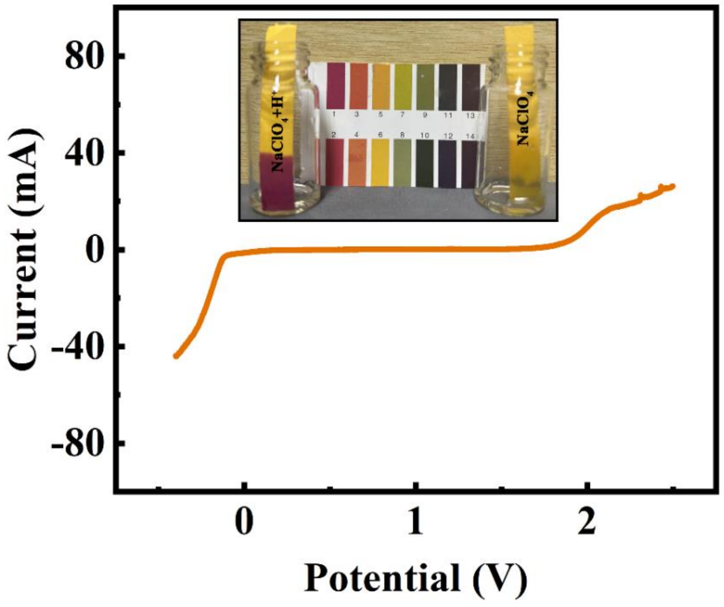

Figure S4. Linear sweep voltammetry of H^+ + $NaClO_4$ electrolyte. Scan rate: 5 mV s^{-1}. Insert graph shows the pH test results of $NaClO_4$ and H^+ + $NaClO_4$ electrolyte. The H^+ + $NaClO_4$ electrolyte is strongly acidic while the $NaClO_4$ electrolyte is alkalescence.

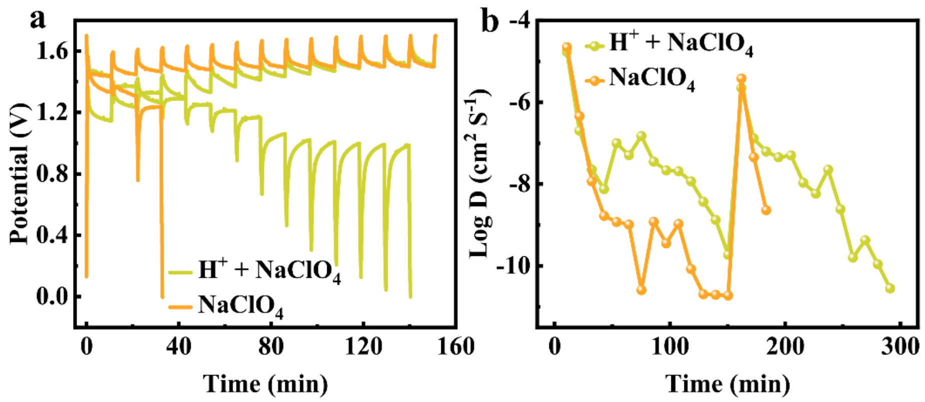

Figure S5. GITT curves of graphite electrodes in H^+ + $NaClO_4$ and $NaClO_4$ electrolytes and the corresponding diffusion coefficients.

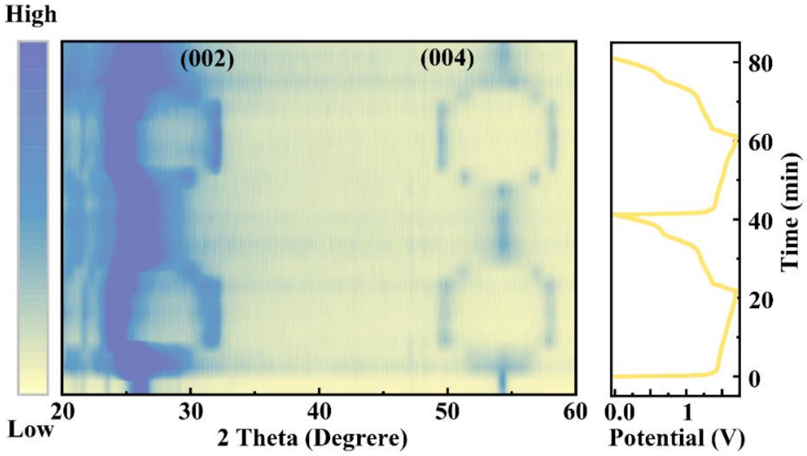

Figure S6. *In-situ* XRD analysis of graphite structure in first two cycles.

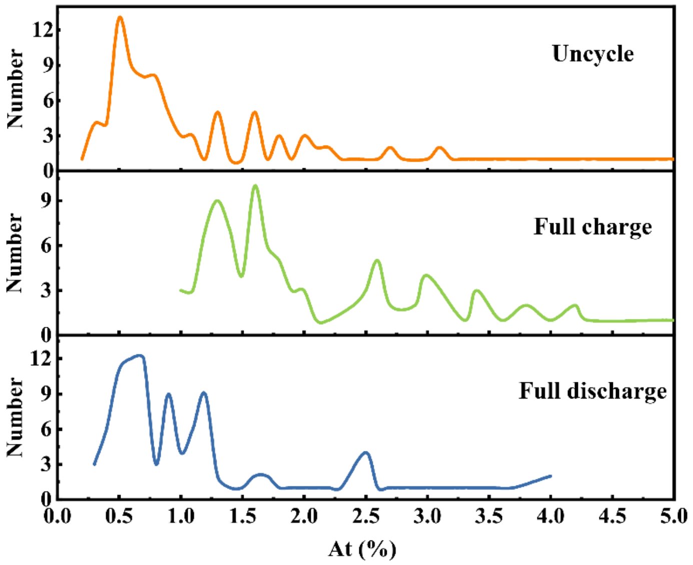

Figure S7. The variation of Cl element content during ClO_4^- (de)intercalation.

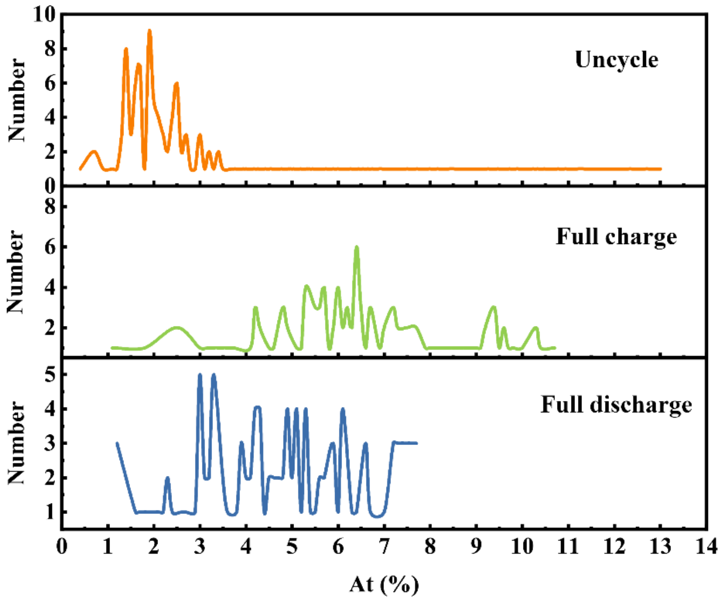

Figure S8. The variation of O element content during ClO_4^- (de)intercalation.

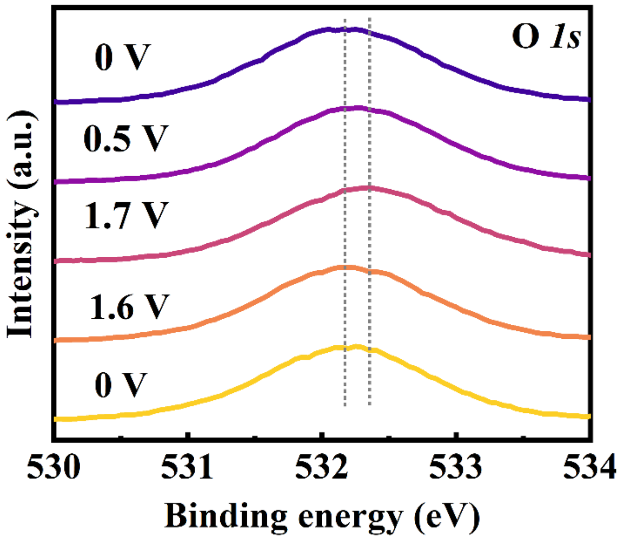

Figure S9. XPS spectra of O *1s* at different potentials.

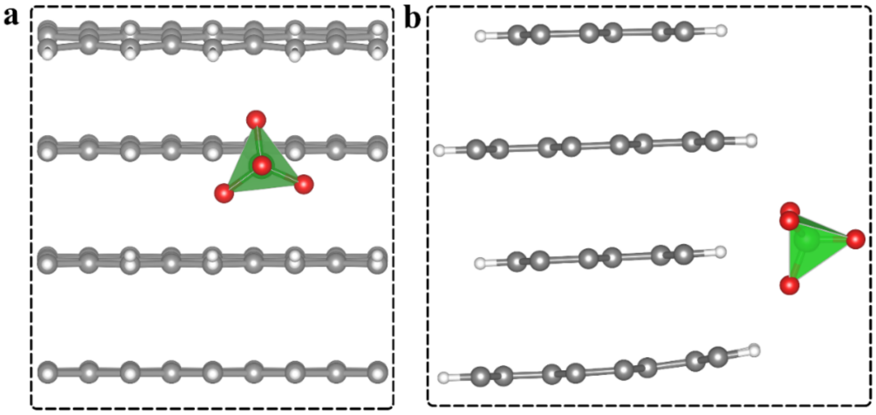

Figure S10. Adsorption mode of ClO_4^- on the edge of graphite planes.

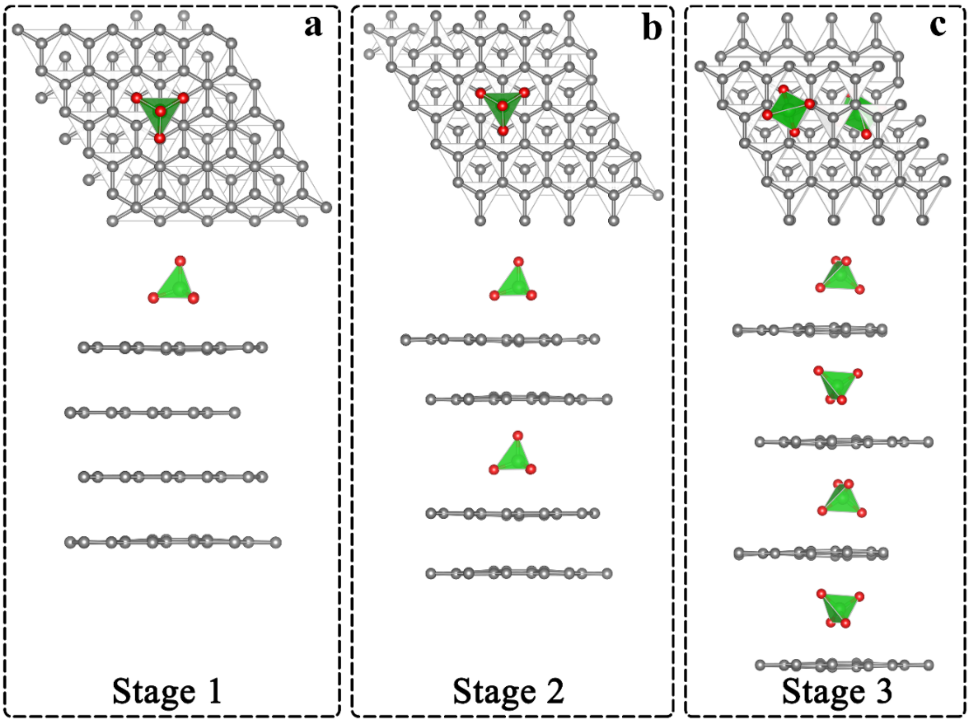

Figure S11. The top and main views of intercalation at different stages.

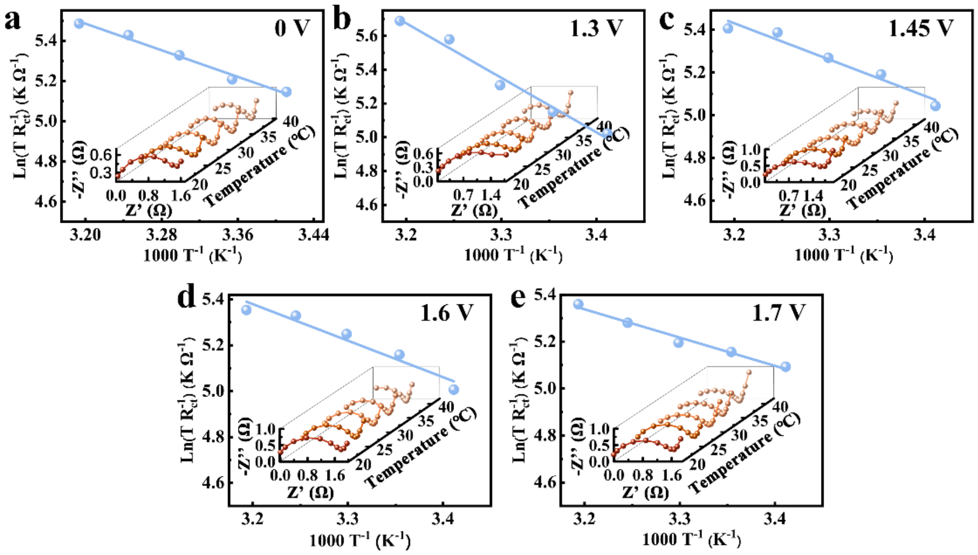

Figure S12. Electrochemical impedance spectroscopy curves during ClO_4^- intercalation at different temperature.

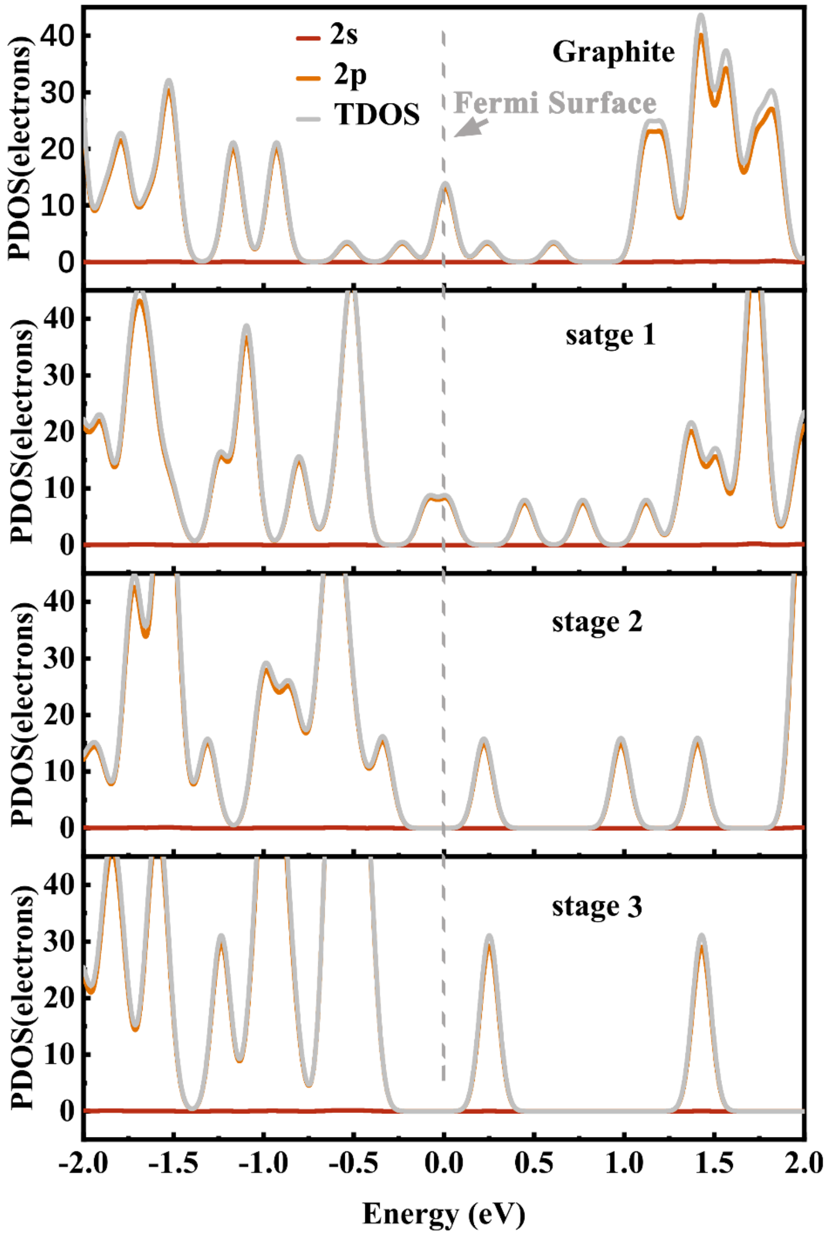

Figure S13. PDOS of different stages.

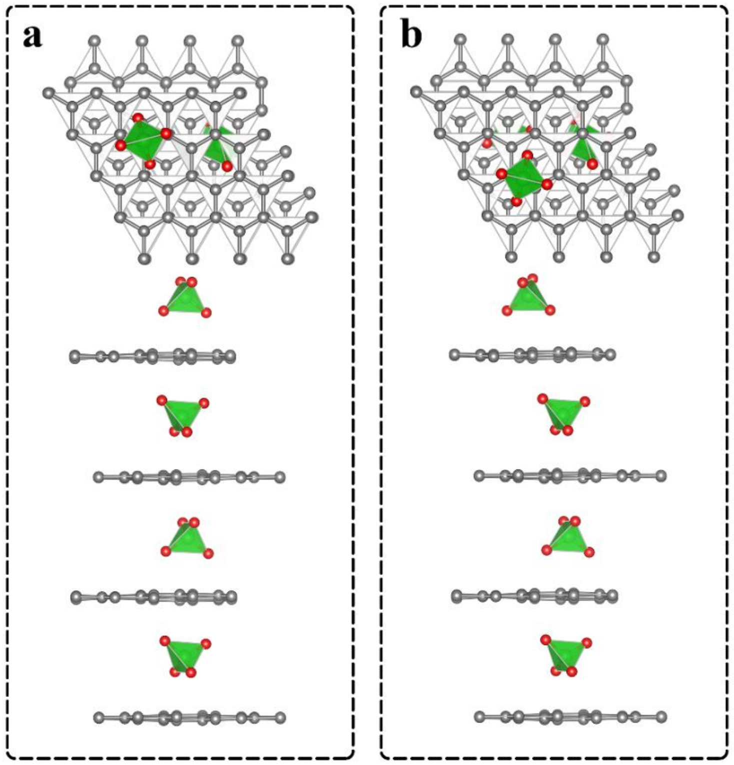

Figure S14. Diffusion mode of ClO_4^- in Stage 3.

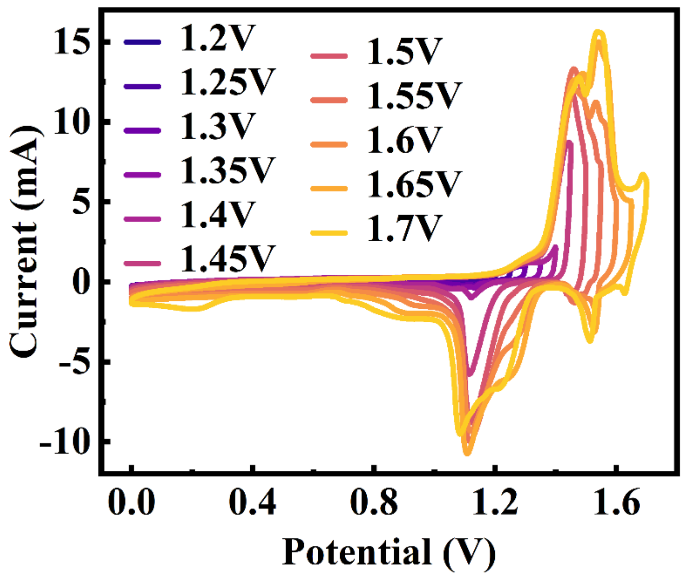

Figure S15. CV curves of ClO_4^- (de)intercalation in graphite with different upper cut-off voltage. Scan rate: 2 mV s^{-1}.

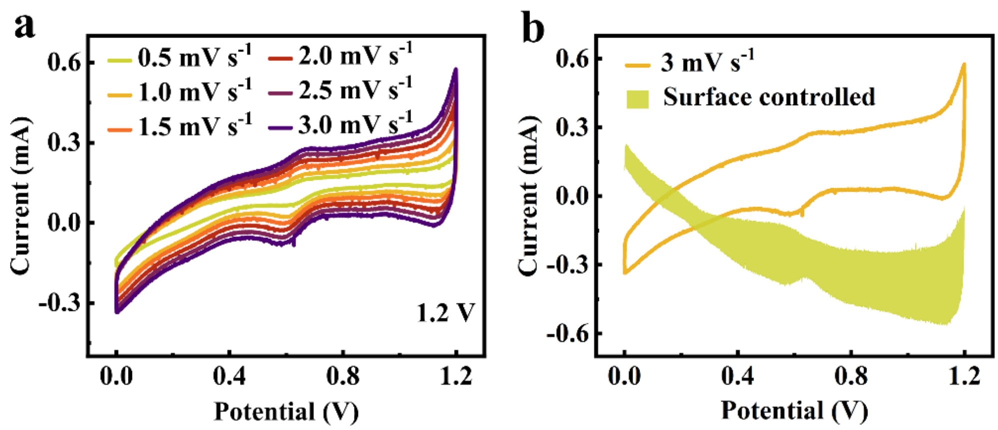

Figure S16. Surface-controlled with the upper cut-off voltage of 1.2 V.

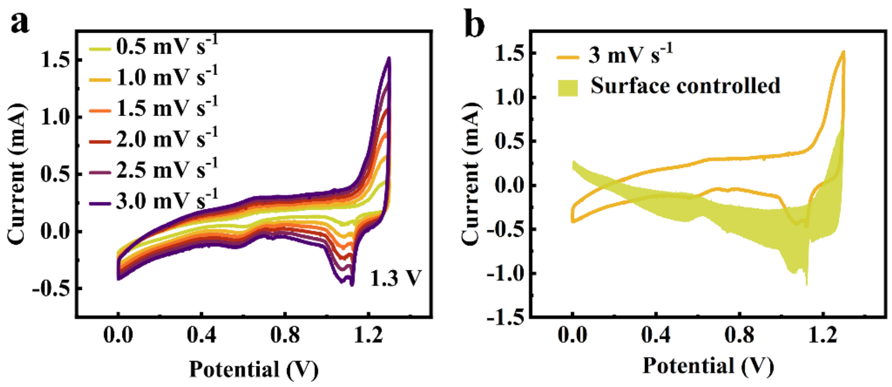

Figure S17. Surface-controlled with the upper cut-off voltage of 1.3 V.

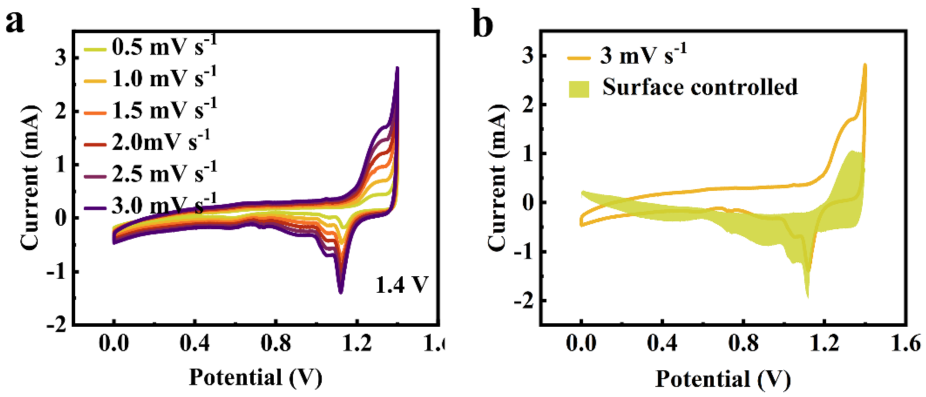

Figure S18. Surface-controlled with the upper cut-off voltage of 1.4 V.

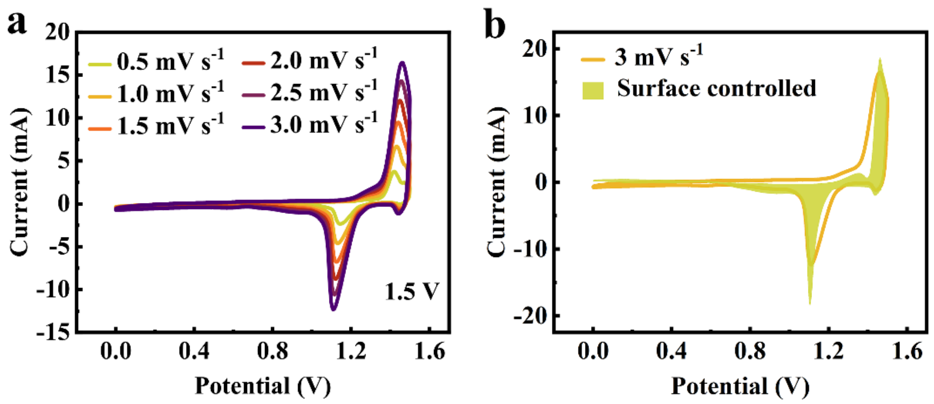

Figure S19. Surface-controlled contribution with the upper cut-off voltage of 1.5 V.

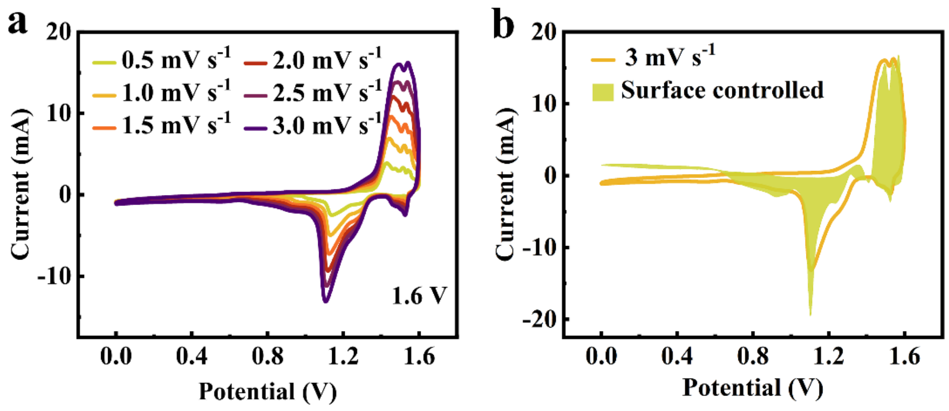

Figure S20. Surface-controlled with the upper cut-off voltage of 1.6 V.

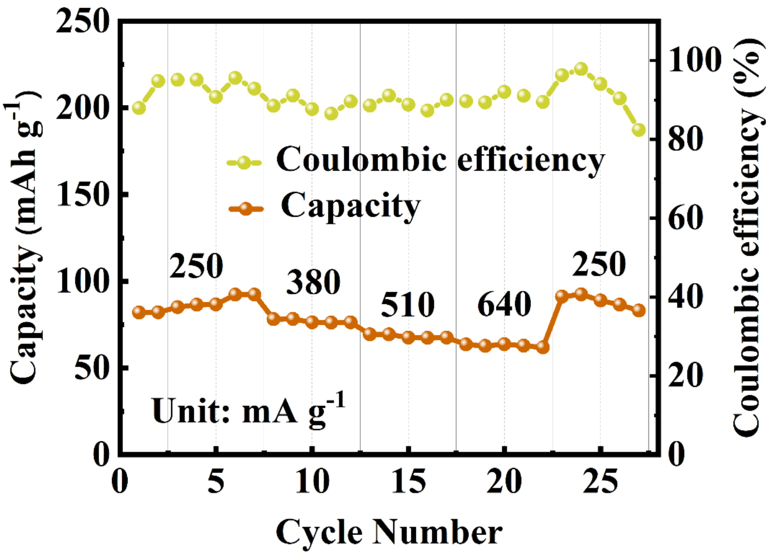

Figure S21. Rate capability and efficiency of ClO_4^- (de)intercalation in graphite in H^+ + $NaClO_4$ electrolyte.

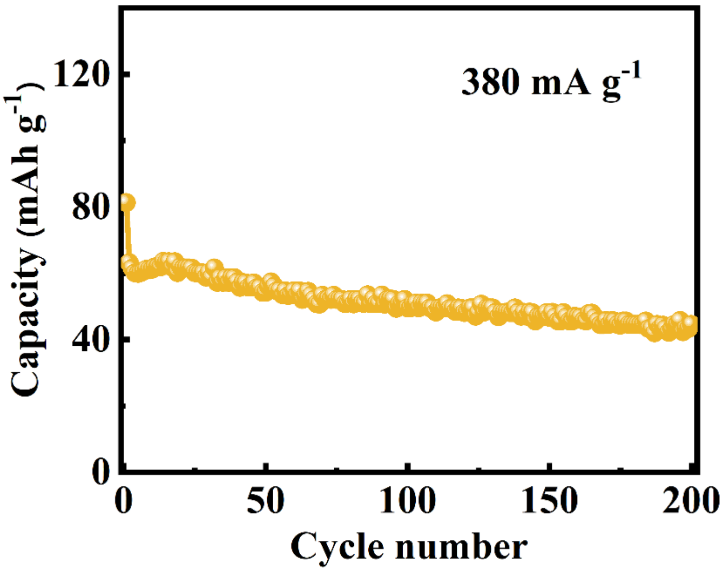

Figure S22. Cyclability of ClO_4^- (de)intercalation in graphite in H^+ + $NaClO_4$ electrolyte.

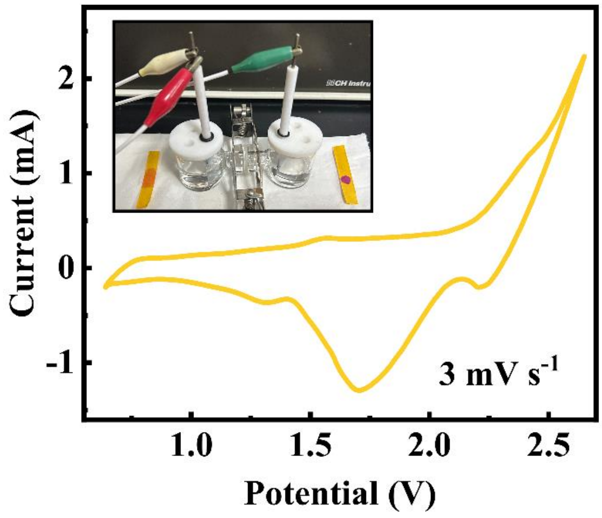

Figure S23. CV curve of Zn-graphite battery at the scan rate of 3 mV s^{-1}. The catholyte and anolyte are $NaClO_4$ + $HClO_4$ and $NaClO_4$ + $Zn(CF_3SO_3)_2$, respectively, separated by an AMV anion exchange membranes. Through pH test paper shown that the catholyte in the positive chamber is strongly acidic while the anolyte in the negative chamber is weakly acidic.

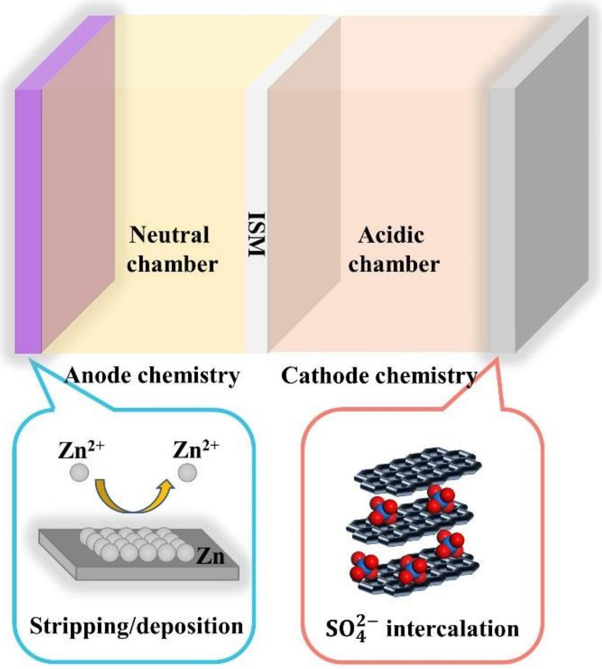

Figure S24. The illustration of the decoupled Zn-graphite battery with SO_4^{2-} intercalation.

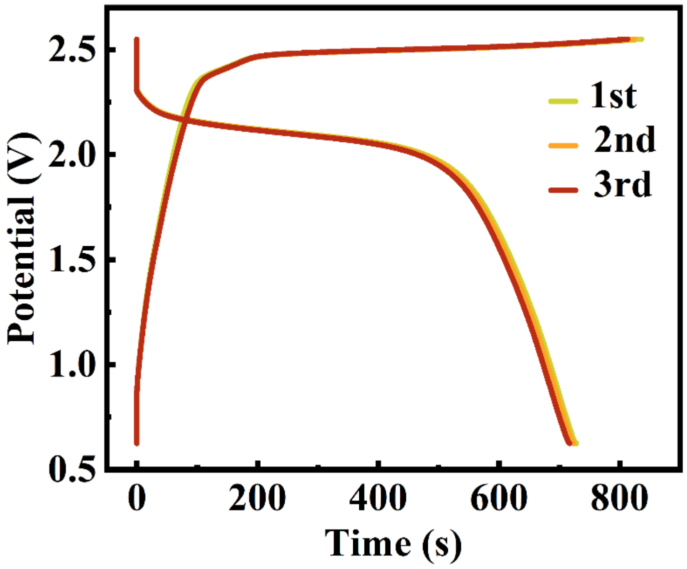

Figure S25. The charge/discharge profiles of Zn-graphite battery with SO_4^{2-} intercalation at the current density of 100 mA g^{-1}

Tables:

Table S1. The energy of ClO_4^- in different structures

	Gcorr (hartree)	E_DFT	G

		(hartree)	(hartree)
ClO_4^- (gas)	–0.01342	–760.939	–760.9521822
ClO_4^--W15 (SMD)	0.308535	–1908.26	–1907.948746
W15 (SMD)	0.305994	–1147.22	–1146.911702

[a] 1 hartree = 2625.5 kJ mol^{-1} = 27.21 eV mol^{-1} = 627.51 kcal mol^{-1}

Table S2. The variation of a, b and c parameters of graphite during ClO_4^- intercalation

	a (Å)	b (Å)	c (Å)
Graphite	9.864527	9.864527	13.600365
Stage1	9.856926	9.85695	16.823069
Stage2	9.849435	9.849277	19.698605
Stage3	9.836164	9.847571	26.42576
